# Unveiling patterns and sociodemographic characteristics of drug trafficking in Dhaka city, Bangladesh

**DOI:** 10.1371/journal.pone.0349429

**Published:** 2026-08-10

**Authors:** Rokonuzzaman, Md Mahdi Hasan, Samia Akter, Md. Alamin, Gareth J. Hollands, Md Shafayat Hossain

**Affiliations:** 1 Department of Pharmacy, Manarat International University, Dhaka, Bangladesh; 2 School of Pharmacy, Changzhou University, Wujin District, Jiangsu Province, P.R. China; 3 MATS, Sirajganj, Bangladesh; 4 Deanship of Scientific Research, Imam Mohammad Ibn Saud Islamic University (IMSIU), Riyadh, Saudi Arabia; 5 EPPI Centre, UCL Social Research Institute, University College London, London, United Kingdom; 6 School of Biosciences, University of Birmingham, Edgbaston, Birmingham, United Kingdom; 7 Department of Pharmacy, School of Pharmacy and Public Health, Independent University, Dhaka, Bangladesh; 8 Biomedical Research Foundation, Uttara, Dhaka, Bangladesh; The University of Iowa, UNITED STATES OF AMERICA

## Abstract

**Purpose:**

Drug trafficking poses significant societal challenges globally, with intricate networks spanning production, transportation, and distribution. Bangladesh, being situated between major drug production hubs, faces persistent challenges despite efforts to combat the issue. This research aims to unveil the patterns and characteristics of drug trafficking in Dhaka City, Bangladesh.

**Methods:**

A descriptive survey of 1016 individuals (mean age = 26.7, the majority (55.7%) having no more than primary education) involved in drug trafficking in Dhaka City, Bangladesh, via in-person interviews using a combination of purposive and snowball sampling methods within non-contrived, natural settings and questionnaires utilizing quantitative assessments. The survey was supplemented by a broader contextual analysis of international secondary data sources concerning drug trafficking networks.

**Results:**

Sociodemographic analysis revealed a diverse profile among traffickers but notably included the significant involvement of women and children aged 15 and under. Incidence of street-living and sex work was substantial in the sample. At the same time, levels of educational attainment were generally low, and coercion was cited as a critical factor in involvement in trafficking by women and children. Heroin, weed/marijuana, and yaba (a mixture of methamphetamine and caffeine) were found to dominate trafficking into Dhaka, with specific localities functioning as key hotspots.

**Discussion:**

The study highlights the complex dynamics of drug trafficking in Bangladesh and a concerning trend of active involvement among vulnerable demographic groups. The findings underscore the urgency of a targeted and multi-pronged holistic approach to intervention. This should aim to integrate community-based strategies to address underlying socio-economic factors with community-oriented law enforcement efforts to disrupt the long-term development and ongoing activity of trafficking networks, accompanied by regional and international cooperation.

## Introduction

Drug trafficking is a multifaceted global issue that presents significant challenges to law enforcement agencies, policymakers, and societies worldwide [1]. Defined as the illegal trade of narcotics, it involves intricate networks spanning production, transportation, and distribution [2]. It mirrors high and growing global prevalence of drug use, with the United Nations Office on Drugs and Crime (UNODC) World Drug Report 2025 estimating that 316 million people worldwide had used drugs in the past year, representing approximately 6% of the global population aged 15–64 years [3,4]. UNODC systematically monitors and researches illicit drug markets to understand their dynamics [3]. For example, it has been determined that a substantial portion of the world’s heroin, approximately 350–580 tons, originates from Afghan opium [5]. Major trafficking routes, such as the Balkan – traversing Iran, Turkey, Greece, and Bulgaria – and northern routes – typically passing through Central Asian countries such as Tajikistan and Kyrgyzstan, which serve as transit points on the route to Russia- serve as key conduits for heroin, linking Afghanistan to markets in the Russian Federation and Western Europe [6]. By contrast, cocaine predominantly circulates in North America and western and central Europe, regions which together accounted for over 40% of global seizures in 2020, reflecting their role as the principal consumer markets. Cocaine production reached almost 2,000 tons in 2020 and has continued to rise steadily over the past decades [7]. The trafficking of cocaine often involves transportation from Colombia to Mexico or Central America by sea, followed by overland routes to the United States and Canada. In Southeast Asia, countries commonly implement stringent penalties for drug-related offenses due to the prevalence of the ‘golden triangle’ of drug production, historically a global center of opium production. These penalties include lengthy prison sentences and, in some cases, the death penalty [8]. Increasingly this region is witnessing extensive trafficking of synthetic drugs. In 2024, authorities in East and Southeast Asia seized a record 236 tons of methamphetamine, representing a 24% rise from the 190 tons confiscated the year before [9]. These findings are consistent with the World Drug Report 2025, which identified amphetamine-type stimulants (ATS) as the dominant category among synthetic drug seizures globally [4]. An international operation coordinated by the International Criminal Police Organization (INTERPOL) targeting drug trafficking in Southeast Asia resulted in unprecedented seizures of synthetic drugs, with an estimated total value of approximately USD 1.05 billion, highlighting the scale and complexity of regional illicit drug networks [10]. Clearly, widespread drug trafficking of a range of substances is a global issue. It has complex societal determinants and impacts, and frequently intersects with other illicit activities, such as sex work. The exploitation of vulnerable individuals, often coerced into sex work due to drug addiction or financial desperation, underscores the interplay between these phenomena [11]. This article specifically examines these phenomena in Bangladesh. Bangladesh is situated between the Golden Triangle and the Golden Crescent and has emerged as a critical transit point for international drug smugglers. Despite not sharing direct borders with Laos or Thailand, its proximity to Myanmar renders it vulnerable to trafficking routes emanating from the Golden Triangle [12]. Yaba (a mixture of methamphetamine and caffeine) and Phensedyl (a pharmaceutical cough syrup combining codeine and chlorpheniramine) continue to flood the market in Bangladesh, exacerbated by Myanmar’s failure to curb yaba production and India’s increased Phensedyl smuggling, reflected in a rising number of seizures by the authorities. Drug networks are known to distribute yaba from Myanmar into Bangladesh through India’s Mizoram, Meghalaya, and Assam. They enter Bangladesh via Zakiganj in Sylhet. Despite increased vigilance in Cox’s Bazar, this route offers less risk to traffickers. Another route via Teknaf-Kuakata was also utilized previously [13]. More recently, drugs like crystal meth pose emerging threats [14].

The scale of trafficking in Bangladesh is reflected in the most recent national data published by the Department of Narcotics Control (DNC), Bangladesh (2023–2024). This reported 20,704 cases involving 21,924 accused individuals, along with the seizure of, for example 3.7 million yaba tablets, 25.5 kg of heroin, 8.5 kilograms of cocaine, and 8,146 kilograms of cannabis [15].

The societal impact of such proliferation of drugs on the Bangladeshi population is considerable. For example, it has been estimated that around 8–8.5 million people in Bangladesh are drug users, primarily young people [16]. The trade is fueled by international drug rings using the country as a transit hub and poses significant challenges to local authorities [11]. In addition to the severe public health and social consequences, the economic burden is equally alarming. The first national estimates of illicit financial flows (IFFs), developed with the support of the United Nations Office on Drugs and Crime (UNODC), indicate that drug trafficking accounted for an annual average of approximately USD 481 million in outward IFFs during 2017–2021, mainly from the trafficking of methamphetamine tablets, heroin, and synthetic opioids such as buprenorphine and Phensedyl [17]. The largest shares of these illicit financial flows were attributed to Phensedyl and yaba/methamphetamine trafficking, followed by buprenorphine and heroin [4].

The evolving socio-economic landscape and demographic shifts within cities like Dhaka create fertile ground for the proliferation of such illicit activities. Against this backdrop, our research endeavors to conduct a comprehensive exploration of drug trafficking routes and patterns in Dhaka City. By adopting a multifaceted analysis that integrates primarily descriptive socio-demographic but also geographic and law enforcement perspectives, we aim to uncover the intricate web of connections that underlie drug trafficking in Bangladesh’s capital. Ultimately, our goal is to inform targeted interventions and policy measures aimed at addressing this pervasive issue.

## Methods

### Research design

**Fig 1:**
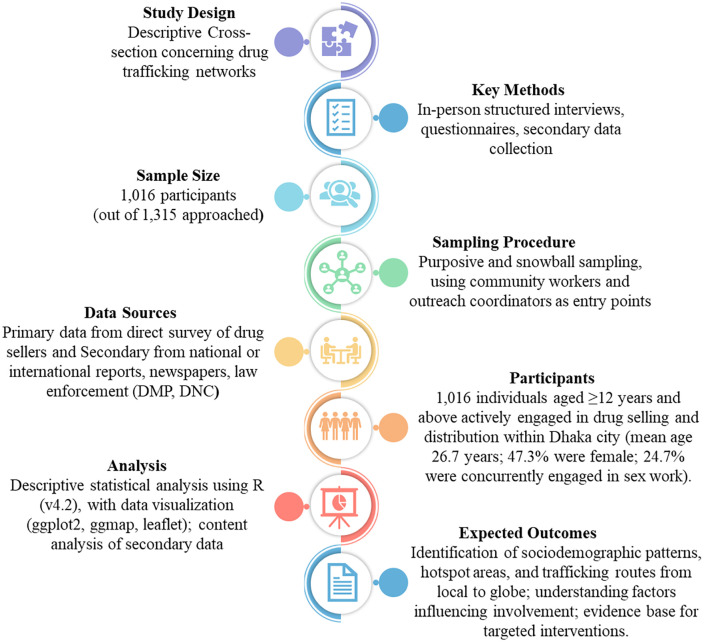
A schematic outline illustrating the study design and data analysis pipeline.

The methodological approach employed in this study aimed to provide a comprehensive understanding of drug trafficking dynamics within Dhaka city. Primarily, this was conducted through a descriptive cross-sectional survey, directly engaging drug sellers (Fig 1). This survey was supplemented with insights derived from a broader contextual analysis of international secondary data sources concerning drug trafficking networks.

### Study site and duration

The study was conducted in Dhaka City, the capital and largest urban center of Bangladesh, from April 23, 2023, to February 5, 2024. Dhaka was selected due to its role as a central hub for drug distribution and its high concentration of vulnerable populations such as street children (operationally defined in this study as participants aged 15 years or younger), sex workers, and economically marginalized individuals.

### Sampling and recruitment

We employed a combination of purposive and snowball sampling to survey individuals involved in drug selling in Dhaka. Purposive sampling was used to identify initial key informants, being individuals with established connections within neighborhoods and with drug-involved communities (e.g., community workers, outreach coordinators, NGO staff). These initial contacts were established through a combination of local knowledge and insights from past community-focused studies, as well as consultations with experts who had previously conducted research in these areas. These informants then referred other individuals, initiating snowball sampling of participants [18], which is widely used in studies of hidden or hard to access populations. Participants recruited were asked to refer others they knew who were involved in similar activities, thereby expanding our network of potential participants [19,20]. Random sampling was not feasible due to the illicit and concealed nature of the drug-related activity we were focusing on. This strategy enabled access to a diverse sample of participants across different subgroups and geographic hotspots (Fig 1). An a priori sample size calculation was not conducted due to the hidden and illicit nature of the study population, the related absence of reliable prevalence estimates, and the infeasibility of probability-based sampling. The study was therefore designed as an exploratory descriptive survey, with recruitment guided by feasibility, access, and safety considerations within the available study period. The recruitment process involved members of the research team initially approaching potential participants to determine whether they were willing to answer questions on a range of issues, including drug trafficking, before revealing that this was for a research study focused on drug trafficking. Individuals were then given the opportunity to ask questions and provide consent to participate.

### Inclusion and exclusion criteria

Participants were eligible for inclusion if they were aged 12 years or older, were currently involved in the sale or distribution of drugs within Dhaka city at the time of recruitment, and were willing to provide informed consent to participate in the study (see ‘Ethical Considerations’ for further details). For the purposes of analysis, the term child/street child was operationally defined as participants aged 15 years or younger; participants aged 16–17 years were not included in this group and instead were categorized within older age categories. Individuals were excluded from the study if they were not involved in selling or distributing drugs within Dhaka city, provided insufficient or incomplete data (e.g., questionnaire responses necessary for analysis were not given) during the data analysis process, or had previously participated in this study (Fig 1). To prevent duplicate participation, each participant was assigned a unique study recruitment ID code linked to strictly maintained logbooks, and the field team cross-checked all referrals before enrollment. Of 1,315 individuals approached, 299 were excluded based on these criteria, resulting in a final sample of 1,016 participants who completed data collection and were included in analyses (Fig 1).

### Data collection tools and procedures

For the descriptive survey, the structured questionnaires that were used for data collection focused on a range of aspects of drug selling within Dhaka city. Principally, participants were asked about their sociodemographic characteristics (gender, age, living conditions, level of education, involvement in sex work) and the types of drugs they sold (combinations of weed/marijuana, yaba, and heroin being identified), and the most common geographic areas in which they sold these. Participants’ living conditions were categorized as living on the street, with their families, in shared accommodation (known as a ‘mess’ in Bangladesh), or being homeless or having no fixed accommodation (referred to as ‘floating’ in Bangladesh). Additional sociodemographic factors examined included perceptions of employment, education, and economic opportunities. A full version of the survey items used is provided in the supplementary material (S1 Appendix.).

Secondary data from scientific journals and recently published national newspapers were identified to gather information on international drug trafficking routes relevant to Dhaka city. These sources and the information they specifically provided about local and regional routes are collected in Supplementary S2–S4 Appendix. First, we gathered information on drug trafficking pathways from multiple sources, which were then categorized into two components: (i) the geographic routes through which drugs enter and transit Dhaka, and (ii) the primary drug types associated with each route. We identified several border districts, including Satkhira, Jashore, Chuadanga, Meherpur, and Rajshahi, along with corresponding vulnerable points and nearby areas along the Indian border. Broadly, we divided the national landscape into western, eastern, and northern regions, along with their respective country borders and crossing points. After identifying national and international routes, we plotted them on a map to track the drug trafficking routes (Fig 1). This process was led by one author (R) and checked by another author (MM). Any uncertainties or disagreements were discussed and resolved by consensus.

To complement this secondary data collection on trafficking routes, we also sought assistance from resident hotel staff to obtain relevant local insights and information on drug trafficking networks, as well as from publicly available sources and official documents of the Dhaka Metropolitan Police (DMP) and the Department of Narcotics Control (DNC). In Bangladesh, resident hotels, especially those near transport hubs and/or frequented by international travelers, often serve as informal points of contact or hubs for illicit activities. Staff working in such environments commonly encounter or observe suspicious activity, behaviors, or patterns linked to drug trafficking operations and may share and discuss this information with colleagues. These insights provided a unique and rarely elicited perspective on trafficking routes and practices that may not be accessible through law enforcement data alone. The information gathered from hotel staff was instrumental in cross-referencing data collected from publicly available DMP and the DNC sources. Informal discussions with hotel staff were not treated as formal qualitative interview data and were used solely to support contextual interpretation and cross-verification of secondary data sources (S2–S5 Appendix.).

### Data analysis

For the primary descriptive survey of individuals involved in trafficking, descriptive data analysis was performed using R (Version 4.5.1) for additional statistical analysis and R packages including “ggplot2,” “ggmap,” and “leaflet” for data visualization (Fig 1). The supplementary research involved content analysis of contextual information from a range of data sources.

### Ethical considerations

All participants consented to participate in the interviews after being fully informed of the research’s purpose and risks and having the opportunity to ask questions. For participants under 18 years old, prior verbal permission from parents or legal guardians, and verbal assent were obtained before they participated in an interview session. Due to ethical sensitivity and concerns for participant safety, gender identity information was not collected from participants aged 15 years or younger. Due to the sensitive, illicit nature of the research topic, it was not possible to obtain written informed consent from the participants; instead, verbal consent was obtained. Many respondents expressed concerns regarding confidentiality, legal implications, and personal safety. Therefore, verbal informed consent was obtained after clearly explaining the purpose of the study, ensuring voluntary participation, and guaranteeing anonymity and confidentiality of their responses. Ethical approval was obtained from the Ethical Approval & Clearance Committee for research of the Department of Pharmacy, Manarat International University (Approval No.: MIU36B.Pharm007) in accordance with the Declaration of Helsinki.

## Results

### Sociodemographic characteristics of drug trafficking participants

Females and males were both substantially involved in drug trafficking (Table 1). Notably, while the highest percentage of local traffickers (35.6%) were within the 26–35 years age category, children aged 15 or under years comprised a substantial proportion (27.4%). In terms of living conditions, although 37% lived in family settings, about 29% of traffickers lived on the streets, while a further 10% defined themselves as homeless or floating (Table 1). Nearly 56% of participants had attained only primary-level education or had received no formal education, and almost a quarter of participants (all female) described themselves as being involved in sex work (Table 1). Overall, while in some respects, there was a diverse sociodemographic profile among participants, this was marked by the significant presence of multiple factors commonly linked to deprivation and disadvantage.

**Table 1:** Sociodemographic characteristics of participants (*n* = 1016).

Variable	Category	No. of participants	Percentage
**Gender**	Male	257	25.3%
	Female	481	47.3%
	Not collected#	278	27.4%
**Age** Mean = 26.7 SD = 11.9 SEM = 0.4	≤15	278	27.4%
	16–25	183	18%
	26–35	362	35.6%
	36–45	133	13%
	≥46	60	6%
**Living Conditions**	Street	291	28.6%
	With Family	378	37.2%
	Mess/Shared accommodation	241	23.7%
	Floating/Homeless	106	10.4%
**Education**	Primary Education or below	566	55.7%
	Below SSC*	253	25%
	Below HSC**	179	17.6%
	Honors	18	1.8%
**Sex work**	Sex worker/sex work involvement	251	24.7%
	Do not identify as sex worker/ no sex work involvement	765	75.3%

**#**Not collected from child participants aged ≤15 years; *SSC: Secondary School Certificate; **HSC: Higher Secondary Certificate

Gender data were not collected from participants aged ≤15 years due to ethical considerations. In this study, ‘child’ or ‘street child(ren)’ refers to participants aged 15 years or younger reflecting differences in data collection for this group due to ethical considerations as well as local contextual usage of these terms.

### Involvement of population subgroups in the trafficking of drug types

The study’s findings highlighted a concerning trend of drug involvement among vulnerable demographics in Dhaka city (Table 2). Street children (those aged 15 or under) were most involved in the selling of yaba (and also notably substantially, heroin), while men (who were generally young, all being under the age of 26–35) were most involved with the selling of weed, as well as similarly of yaba. Women not involved in sex work were most associated with the sale of heroin and weed, whilst those involved in sex work most commonly sold yaba (Table 2). These patterns underscore the likely need for targeted preventive measures and support systems in relation to clusters of particular drugs and population subgroups involved in trafficking.

**Table 2:** Drug trafficking patterns across population groups.

Drug(s) involved with	Child (≤15 years) Participants (%)	Men not identifying as sex worker^#^ Participants (%)	Women not identifying as sex worker Participants (%)	Women identifying as sex worker Participants (%)
Weed	71 (25.5%)	74 (28.8%)	57 (24.8%)	51 (20.3%)
Yaba	80 (28.8%)	72 (28%)	62 (26.9%)	71 (28.3%)
Weed and Yaba	56 (20.1%)	59 (22.9%)	48 (20.9%)	65 (25.9%)
Weed and Heroin*	71 (25.5%)	52 (20.2%)	63 (27.4%)	64 (25.5%)
Total	278	257	230	251

*We identified no individuals who sold only heroin and none who sold yaba and heroin; #No men identified as sex workers. Age-based categories reflect the study’s operational definitions as described in the Methods section.

### Self-reported reasons for involvement in drug trafficking

Participants reported several socio-economic and contextual reasons for their involvement in drug trafficking activities that differ by demographics (Table 3). For children, the most common reason given was lack of employment opportunities, as it was for women, whether identifying as sex workers or not. Importantly, for these groups of children and especially women, coercion was an important self-reported reason for involvement in drug trafficking. For men, financial insecurity and a lack of economic status or opportunities was regarded as the most frequent explanations (Table 3). It merits emphasis that these findings are descriptive, reflecting participants’ self-reported reasons for involvement in drug trafficking and cannot be assumed to represent causal or statistically verifiable relationships.

**Table 3:** Self-reported reasons for involvement in drug trafficking.

Demographic group	Factors	Percent
**Child (≤15 years)** **(n = 278)**	Lack of employment opportunities	54.4%
	Lack of education	22.3%
	Finances/lack of economic security or status	20.3%
	Coercion in drug trafficking	3.0%
**Men not identifying as sex worker (n = 257)**	Lack of employment opportunities	22.5%
	Lack of education	35.3%
	Finances/lack of economic security or status	42.2%
	Coercion in drug trafficking	0.0%
**Women not identifying as sex worker (n = 230)**	Lack of employment opportunities	33.2%
	Lack of education	27.5%
	Finances/lack of economic security or status	20.0%
	Coercion in drug trafficking	19.3%
**Women identifying as sex worker (n = 251)**	Lack of employment opportunities	42.1%
	Lack of education	17.6%
	Finances/lack of economic security or status	20.3%
	Coercion in drug trafficking	20.0%

### Local distribution of drug trafficking in Dhaka city

**Fig 2:**
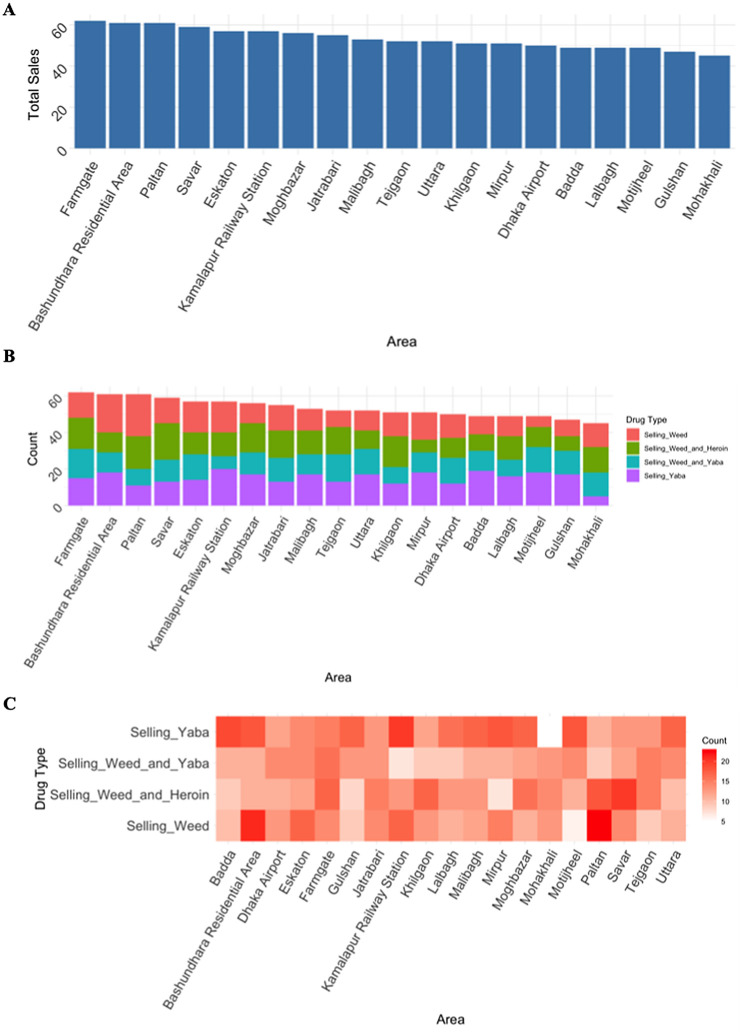
(A) Bar chart for total drug sales by area, (B) Stacked chart for drug sales by type and area (C) Heatmap of drug sales by type and area. The maps were self-designed by the authors using R packages (‘ggplot2’, ‘ggmap’, and ‘leaflet’) with OpenStreetMap base map data.

Of 1016 surveyed participants across Dhaka city, 253 (24.9%) individuals were involved in selling only weed. This single-drug activity was reported as most prevalent in Paltan (9.1%) and Bashundhara residential areas (8.3%) (Table 4 and Fig 2). Similarly, 285 (28.1%) participants were found to be selling only yaba, with the highest concentrations in Kamalapur Railway Station (7.0%) and Badda (6.7%) (Table 4 and Fig 2). 228 (22.4%) participants were involved in selling both weed and yaba, most commonly in Farmgate (7%) and Tejgaon (6.6%). Finally, although we did not identify any sellers who only sold heroin, 250 (24.6%) participants were involved in selling both weed and heroin, with the highest activity observed in Savar (8%) and Paltan (7.2%) (Table 4 and Fig 2).

**Table 4:** Numbers of participants involved in selling main categories of drugs by the areas where they are most active.

Area where participant most active	Selling weed Participants (%)	Selling yaba Participants (%)	Selling weed and yaba Participants (%)	Selling weed and heroin* Participants (%)
Savar	14 (5.5%)	13 (4.6%)	12 (5.3%)	20 (8%)
Mirpur	15 (5.9%)	18 (6.3%)	11 (4.8%)	7 (2.8%)
Mohakhali	13 (5.1%)	5 (1.7%)	13 (5.7%)	14 (5.6%)
Motijheel	6 (2.4%)	18 (6.3%)	14 (6.1%)	11 (4.4%)
Badda	10 (4%)	19 (6.7%)	11 (4.8%)	9 (3.6%)
Bashundhara residential area	21 (8.3%)	18 (6.3%)	11 (4.8%)	11 (4.4%)
Dhaka airport	13 (5.1%)	12 (4.2%)	14 (6.1%)	11(4.4%)
Eskaton	17 (6.7%)	14 (4.9%)	14 (6.1%)	12 (4.8%)
Farmgate	14 (5.5%)	15 (5.3%)	16 (7.0%)	17 (6.8%)
Gulshan	9 (3.5%)	17 (6.0%)	13 (5.7%)	8 (3.2%)
Jatrabari	14 (5.5%)	13 (4.6%)	13 (5.7%)	15 (6%)
Kamalapur Railway station	17 (6.7%)	20 (7.0%)	7 (3.1%)	13 (5.2%)
Khilgaon	13 (5.1%)	12 (4.2%)	9 (3.9%)	17 (6.8%)
Lalbagh	11(4.3%)	16 (5.6%)	9 (3.9%)	13 (5.2%)
Malibagh	12 (4.7%)	17 (6.0%)	11 (4.8%)	13 (5.2%)
Moghbazar	11 (4.3%)	17 (6.0%)	12 (5.3%)	16 (6.4%)
Paltan	23 (9.1%)	11 (3.8%)	9 (3.9%)	18 (7.2%)
Tejgaon	9 (3.6%)	13 (4.6%)	15 (6.6%)	15 (6%)
Uttara	11 (4.3%)	17 (6.0%)	14 (6.1%)	10 (4%)
**Total**	253 (100%)	285 (100%)	228 (100%)	250 (100%)

* We identified no individuals who sold only heroin and none who sold yaba and heroin

### Regional and domestic trafficking routes

This section draws primarily on secondary data sources (including scientific journals, national newspaper reports, and official documents by DMP and DNC) supplemented by primary data from informants (see also Supplementary Tables: S2–S4 Appendix. for additional detail about where information documenting trafficking routes was sourced).

**Fig 3:**
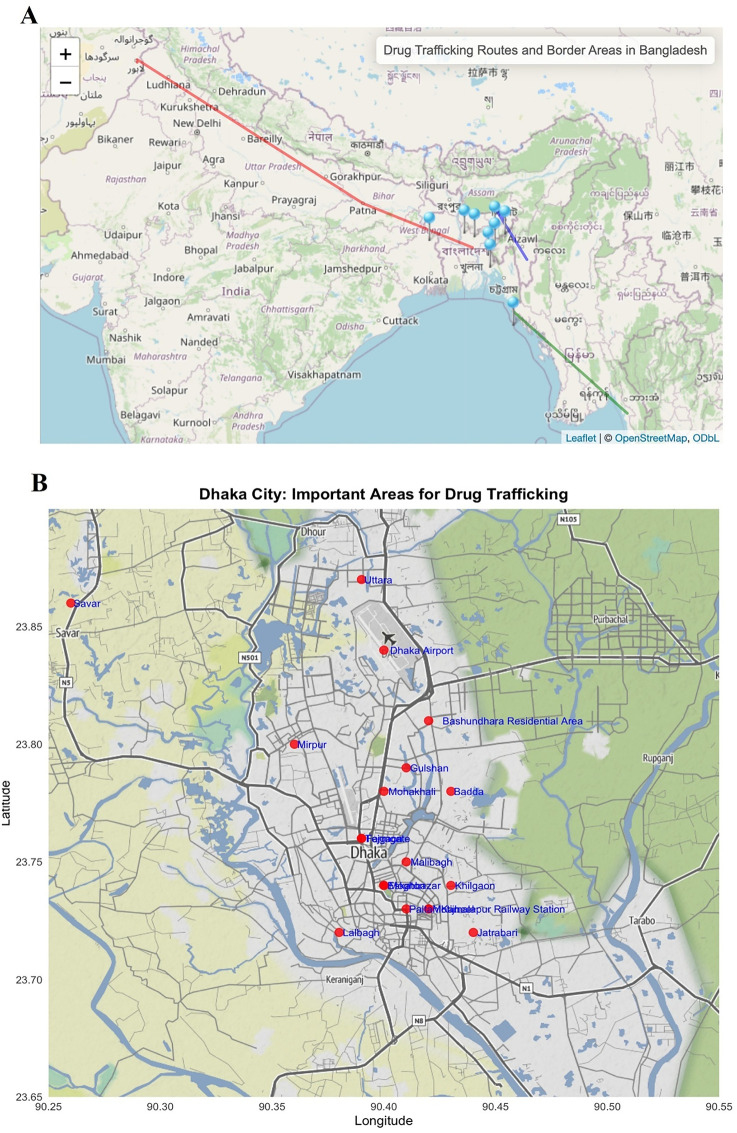
(A) Drug trafficking routes and border areas in Bangladesh. Red colored lines indicate the route of Pakistan-Lahore-Wagah-Patna-Bangladesh. Blue colored lines indicate the route of India-Mizoram-Meghalaya-Bangladesh. Green colored lines indicate the route of Myanmar -Teknaf-Bangladesh. Blue pins indicate entry points into Bangladesh. (B) Important areas for drug trafficking in Dhaka City (indicated by red dots). Map data © OpenStreetMap contributors, licensed under the Open Database License (ODbL) and Leaflet (BSD 2-Clause License). See https://www.openstreetmap.org/copyright and https://github.com/Leaflet/Leaflet/blob/main/LICENSE for details.

International and regional routes identified from secondary literature indicate that heroin trafficking into Dhaka primarily originates from Pakistan, particularly Lahore, following a route through Wagah (Pakistan) and Patna (India) before eventually entering Bangladesh (Fig 3). Based on newspaper and official report analysis, upon its arrival in Dhaka, we suggest that the distribution network extends to various localities such as Savar, Mirpur, Mohakhali, and Motijheel, among others. Similarly, secondary data identifies two main trafficking routes for weed (marijuana) into Dhaka: one entering from India through Brahmanbaria (Bangladesh) and the other through Cumilla (Bangladesh), with both routes converging in Dhaka (Fig 3). The distribution of weed then spreads across the same localities as heroin. The most prominent routes for yaba (methamphetamine) trafficking, as documented in secondary sources, originate from Myanmar, then passing through Mizoram (India) and Meghalaya (India), with multiple routes going through Sylhet (Bangladesh) and Sunamganj (Bangladesh) before reaching Dhaka city (Fig 3). The identified routes for yaba (methamphetamine) originating from Myanmar and traversing through India’s Mizoram and Meghalaya states represent specific localized segments of the broader Golden Triangle trafficking network [4,12,13]. These pathways illustrate how production hubs in the Golden Triangle region directly funnel illicit substances into the Dhaka market [4,12]. Other routes include entry points originating from India through various districts of Bangladesh, such as Jamalpur, Chapainawabganj, Cumilla, and Mymensingh. Local distribution patterns in Dhaka for yaba, cross-verified through informant reports were found to be similar to those for heroin and weed, targeting similar localities for distribution (Fig 3).

## Discussion

This study of drug trafficking in Dhaka city, Bangladesh, highlights its complex dynamics and a concerning trend of active involvement among vulnerable demographic groups. This demands a critical analysis that integrates our findings but also emphasizes their broader implications, including challenges and opportunities for intervention. Below, we outline four key sets of implications.

### Sociodemographic vulnerabilities and the structural drivers of drug trafficking

The participation of young men, women, and socio-economically marginalized groups in the drug trade reflects structural and systemic inequities, including poverty, lack of education, and limited social protections. Such dimensions and expressions of (in)equity rarely function in isolation but instead interact and intersect with one another in complex ways [21]. These findings mirror the “vicious cycle” described in the World Drug Report 2025, whereby socio-economic instability, inequality, and marginalization both contribute to and are exacerbated by drug trafficking and harmful drug use [4]. Socio-economic deprivation is a key driver of involvement in drug trafficking, pushing individuals and families experiencing poverty, unemployment, and social marginalization toward illicit economies as a means of financial survival [2]. The prominent prevalence of females observed in our data is consistent with previous findings about trafficking tending to prey on such socio-economically vulnerable and unprotected people [22]. Similarly, the substantial involvement of children and sex workers observed in this study reflects broader global concerns regarding the exploitation of vulnerable populations within illicit drug markets.

Effective interventions must, therefore, address the underlying socio-economic vulnerabilities that enable and maintain trafficking. For instance, community-based programs offering education, vocational training, and economic incentives have been effective in deterring involvement in illicit activities [2]. Furthermore, specific initiatives for women and children, such as gender-sensitive economic policies and child protection programs, can address the coercion and exploitation prevalent in trafficking networks.

### Geographic concentrations, network dynamics, and community-oriented enforcement

The spatial concentration of trafficking activities in urban hotspots such Jatrabari, Malibagh, Paltan, Bashundhara, Kamalapur, Badda, Farmgate, Tejgaon, and Savar reflects the strategic use of areas that represent a combination of high population densities, economic deprivation, and limited law enforcement presence. These findings align with prior evidence that drug networks thrive in regions with socio-economic and institutional vulnerabilities [23].

Current enforcement strategies often focus on directly reactive measures, such as arrests, which do not reflect and risk overlooking the enduring systemic issues that sustain trafficking networks. To enhance efficacy, interventions should adopt predictive policing and community-oriented approaches that, over the longer term, build trust and foster intelligence-sharing with local populations. Collaborative policing models, which have shown success in other urban contexts, could be adapted to Dhaka’s specific challenges [24].

The observed predominance of single-substance yaba trafficking relative to exclusive either heroin or combined yaba–heroin trafficking may reflect differences in market organization, consumer demand, and trafficking network structures within Dhaka city. Yaba has been widely reported as one of the most prevalent synthetic drugs in Bangladesh and Southeast Asia, particularly among younger populations, contributing to its extensive circulation through localized street-level distribution networks [3,9,15,16]. The yaba trafficking pathways identified in this study through Myanmar and northeastern India may best be understood as localized extensions of the broader Golden Triangle trafficking network, historically associated with methamphetamine production and distribution across Myanmar, Laos, and Thailand [12,13]. These regional trafficking dynamics further reinforce Bangladesh’s vulnerability as a transit and distribution point within interconnected Southeast and South Asian illicit drug markets. By contrast, heroin trafficking may operate through comparatively more restricted or specialized supply chains, potentially explaining why heroin was only identified in combination with weed/marijuana in this study. The absence of reported yaba–heroin co-trafficking may additionally indicate partial segmentation between stimulant- and opioid-oriented trafficking networks, consistent with evidence suggesting that illicit drug markets often function through differentiated organizational structures and distribution channels [22,23]. However, both these findings and their explanations are necessarily tentative and will require further research and corroboration, particularly given the possibility of methodological limitations associated with sampling from hidden populations, including underreporting due to stigma or participant hesitancy [18,20].

Regional shifts in drug markets also indicate emerging threats that may influence future trafficking patterns in Bangladesh. The World Drug Report 2025 notes that reduced opium production in Afghanistan following the 2022 cultivation ban has contributed to concerns regarding opiate shortages and the possible expansion of potent synthetic opioids such as nitazenes as substitutes [4]. These substances are frequently mixed with or misrepresented as heroin and present substantial overdose and public health risks due to their extreme potency. Continuous monitoring of evolving trafficking routes and synthetic drug markets will therefore be essential for Bangladesh and neighboring countries. Further mixed-methods research is needed to determine whether these findings reflect broader trafficking dynamics within Bangladesh.

### Cross-border trafficking and regional and international cooperation

Dhaka’s role as a transit hub for drugs, particularly yaba and heroin, highlights the transnational dimensions of trafficking. Porous borders with Myanmar and India, coupled with fragmented enforcement, exacerbate the problem. This mirrors global trends where weak border controls and inconsistent regional policies create vulnerabilities that traffickers exploit [2]. This suggests that Bangladesh must strengthen its regional cooperation efforts, drawing lessons from successful models such as the Mekong Memorandum of Understanding on Drug Control, which integrates intelligence sharing and joint operations [25]. Bilateral agreements with neighboring countries should prioritize the harmonization of drug policies, enhanced border surveillance, and the establishment of joint task forces.

### Integrative and multi-level approaches

In sum, our findings and their broader implications outlined above underscore the need for a multi-pronged, holistic approach that integrates socio-economic, enforcement, and international collaborative strategies. Isolated interventions, such as solely focusing on reactive enforcement, risk exacerbating vulnerabilities without addressing root causes.

This approach to intervention should be underpinned by restorative justice principles, emphasizing rehabilitation over punitive measures, particularly for participants aged ≤15 years and coerced traffickers. Studies have demonstrated that reintegration programs reduce recidivism and improve long-term outcomes for traffickers and drug users [23]. Furthermore, public education campaigns can play a supportive role in building community resilience and reducing vulnerability to trafficking. From a policy perspective, the results emphasize the need for evidence-based strategies that extend beyond punitive enforcement. Practical measures may include strengthening regular patrols in identified trafficking hotspots, increasing community policing units, and enhancing the role of local government bodies in monitoring vulnerable neighborhoods [26]. Employment generation and rehabilitation services for coerced or vulnerable traffickers can be directly integrated into urban development and social welfare programs. In addition, stronger coordination between law enforcement agencies and community-based organizations can ensure more consistent intelligence-sharing and targeted operations. Such localized actions in Dhaka also align and link with global calls for integrated, multi-sectoral strategies, highlighting the international relevance of key actions of community-oriented enforcement, gender-sensitive interventions, and cross-border cooperation [27]. This has the potential to inform Bangladeshi policymaking but also provide a reference point for global strategies to address drug trafficking in comparable socio-economic contexts.

### Strengths and limitations of the study

This study contributes valuable insights into the sociodemographic and geographic dimensions of drug trafficking in Dhaka, gathering extensive data from hard-to-reach and rarely documented population subgroups. More broadly, it contributes to global knowledge by documenting the intersections of sociodemographic vulnerability, coercion, and geographic concentration. By showing how children, women, and socially marginalized groups are systematically drawn into trafficking networks, the findings add comparative insight to international debates on the structural drivers of illicit activities and economies.

The study has some limitations. Reliance on self-reported data introduces potential biases, including underreporting due to stigma. Additionally, the study’s focus on Dhaka may limit its generalizability to other regions of Bangladesh and to other countries. The absence of a priori sample size estimation is a methodological limitation that may affect the precision and generalizability of the findings. Results should therefore be interpreted with due caution as descriptive and exploratory rather than truly population-representative. Furthermore, data gathered from hotel staff relied on informal, convenience-based discussions rather than structured interviews, making these specific insights susceptible to recall bias, subjective interpretation, and limited representativeness. To mitigate this risk, this information was used strictly for contextual cross-referencing and was rigorously triangulated with official reports, scientific literature, and national newspaper sources. For example, during these discussions/interviews, we asked hotel staff whether they had knowledge of where drug-related activities occur nearby and/or across Dhaka City, who is involved, and the routes and processes through which drugs are transported and sold. Future research should employ a broader set of mixed-methods approaches to enable triangulation of data from a wider range of sources and expand geographic coverage to capture a more comprehensive picture of trafficking dynamics within and beyond Bangladesh.

## Conclusion

These findings highlight the severity and complexity of the drug trafficking situation in Bangladesh and underscore the urgency of a targeted and multi-pronged holistic approach to intervention to address this. This should aim to integrate community-based strategies to address underlying socio-economic factors with community-oriented law enforcement efforts to disrupt the long-term development and ongoing activity of trafficking networks, accompanied by regional and international cooperation. By addressing in tandem persistent underlying systemic vulnerabilities and the activity of trafficking networks, policymakers can develop a more collaborative, sustainable, and humane approach to combating drug trafficking in the longer term and at local, national, and international levels.

### Author agreement statement

It is hereby certified that all authors have reviewed and approved the final version of the manuscript being submitted. Additionally, it confirms that the article represents the original work of the authors, has not been published previously, and is not currently under consideration for publication elsewhere.

## Supporting information

S1 Appendix Survey questionnaires. (DOCX)

S2 Appendix Routes of drug trafficking into Bangladesh: Insights from national newspaper data. (DOCX)

S3 Appendix Vulnerable Points of Trafficking in Bangladesh and India identified from Department of Narcotics Control. (DOCX)

S4 Appendix Major border crossing routes based on Department of Narcotics Control. (DOCX)

S5 Appendix Narcotics Seizures by DNC. (DOCX)
