# Supplementary material for: Unveiling patterns and sociodemographic characteristics of drug trafficking in Dhaka city, Bangladesh
Source: PLoS One. 2026 Aug 10;21(8):e0349429. doi: 10.1371/journal.pone.0349429 (PMC13456272; doi:10.1371/journal.pone.0349429)
Supplement: Supplementary file 1 [file pone.0349429.s001.docx]

**S1 Appendix.** Survey questionnaires.

**Survey Questions**

**Participant Information:**

1. Name of the respondent (Optional)
2. Age (self-reported)
3. Gender (self-reported)

**Social status:**

1. What is your status?

- Child; Adult man; Adult woman
- Identifies as Sex worker/Involved in sex work (vs Not)

**Living arrangements (self-reported):**

1. What is your current living situation?

- Street
- With Family
- Mess/Shared accommodation
- Floating/Homeless

**Educational Background:**

1. Education level (self-reported):

- Primary education or below
- Below Secondary School Certificate (SSC)
- Higher Secondary Certificate or below (HSC)
- Honors

**Drug Sales and Influencing Factors:**

1. Which type of drugs are you involved in selling?

- Weed
- Yaba
- Heroin
- Weed and yaba
- Weed and heroin
- Yaba and heroin
- Other

2. In which area(s) do you sell drugs?
3. What factors influenced your involvement in drug trafficking?

- Lack of employment opportunities
- Lack of education
- Lack of economic opportunities
- Coercion in drug trafficking
