# Supplementary material for: Unveiling patterns and sociodemographic characteristics of drug trafficking in Dhaka city, Bangladesh
Source: PLoS One. 2026 Aug 10;21(8):e0349429. doi: 10.1371/journal.pone.0349429 (PMC13456272; doi:10.1371/journal.pone.0349429)
Supplement: Supplementary file 2 [file pone.0349429.s002.docx]

**S2 Appendix. Routes of drug trafficking into Bangladesh: Insights from national newspaper data.**

| **No.** |  | **Publication Name** | **Author name** | **Route identified** | **Main drugs trafficked** |
| --- | --- | --- | --- | --- | --- |
| 1. |  | The Daily Star | Tasneem Tayeb  22 Nov 2022 | Golden Wedge  Golden Triangle  Golden Crescent  Bangladesh | All kinds of drug especially Yaba. |
| 2. |  | Dhaka Tribune | Probir Kumar Sarker  06 Mar 2024 | Europe & North America  India  Myanmar  Coastal Border  Naf River  Bangladesh  (Entry points)  Europe & North America  Dhaka  (Major drug Market) | Yaba,  Heroin,  Phensedyl (Codeine-based syrup),  Crystal Meth (ICE)  Cocaine, and LSD  Cannabis  Buprenorphine Injection |
| 3. |  | Dhaka Tribune | Tribune Desk  24 Aug 2017, | Myanmar  Rakhine State  Cox's Bazar  Coastal Border & Naf River  (Entry Points)  Bangladesh (Entry points)  Dhaka (Major drug Market) | Yaba |
| 4. |  | Dhaka Tribune | Marin Amin  05 Oct 2023 | Myanmar  Naf River (Entry and distribution)  Bangladesh  Teknaf (RAB-15 Raid and Arrest)  Dhaka (Entry points)  Dhaka (Major drug Market) | Yaba |
| 5. |  | Dhaka Tribune | Nasir Uddin Rocky  08 Feb 2024 | Cox's Bazar Railway Route  Cox's Bazar  Drug Entry Point  Distribution  Chittagong  Railway Surveillance Zone  Dhaka and Other Parts of the country | Yaba and other contraband drugs. |
| 6. |  | Dhaka Tribune | Abdul Aziz  18 Jan 2023 | Palangkhali Union  Border Pillar No 20  Bangladesh-Myanmar Frontier  Dhamankhali Border Area  Cox's Bazar  (Entry Point and Distribution) | Yaba |
| 7. |  | Dhaka Tribune | Imran Hossain  12 Sep 2021 | Myanmar  Naf River  Border Area  Cox's Bazar  Damdamia Nature Park Area  Tulatuli Area  Rajapalang Union  Hnila Union | Yaba |
| 8. |  | The Daily Star | Mohammad Jamil Khan  23 Dec 2019 | Fatehpur Border Point  Bishrosia Village  Panka Union  (Zero Point)  Naf River  (Border Crossing)  Cox's Bazar District  Shibganj Upazila  Chapainawabganj  Sylhet  Jamalpur  Mymensingh  Cumilla | Yaba |
| 9. |  | The Daily Star | Mohammad Jamil Khan  13 Sep 2020 | Rohingya Camps in Myanmar  Naikhyangchhari Upazila  Tombru Border Point  Border Crossings (Rohingya Camps)  Cox's Bazar District  Teknaf and Cox's Bazar Sadar  Ghumdhum Border Point  Naikhyangchhari Border Area  Cox's Bazar-Chattogram  Highway  Chattogram  Bandarban Sadar  Other Districts in  Bangladesh | Yaba |
| 10. |  | The Economic Times | 01 Jul, 2021 | Churaibari in Assam    Bangladesh  Tripura border | Dry Ganja (Weed) |
