# Supplementary material for: Unveiling patterns and sociodemographic characteristics of drug trafficking in Dhaka city, Bangladesh
Source: PLoS One. 2026 Aug 10;21(8):e0349429. doi: 10.1371/journal.pone.0349429 (PMC13456272; doi:10.1371/journal.pone.0349429)
Supplement: Supplementary file 3 [file pone.0349429.s003.docx]

**S3 Appendix.** **Vulnerable Points of Trafficking in Bangladesh and India identified from Department of Narcotics Control.**

| **District** | **Vulnerable points of trafficking in Bangladesh Side** | **Counter areas in Indian side** |
| --- | --- | --- |
| Satkhira | Kaliganj, Debhata, Bhomra, Takiya, Kalaroa, Kakdanga, Plashpur | Hingalgate, Hasnabad, Taki, Basirhat, Swarupnagar, Baduria & adjacent areas of North 24 Parganas |
| Jashore | Benapole, Putkhali, Chowgacha, Narayunpur, Sharsha and adjacent areas. | Chumpakpur, Bongon, Petrapol, Helencha, Amritabazar, Nochhota & adjacent areas of North 24 Pargana, Paschimghata |
| Chuadanga | Kapasdanga Darshana Jiban Nagar | Krishnapur & adjacent areas Naddia, Paschimbangla |
| Meherpur | Dariapur, Buripota, Tetulia, Muhingar | Biranpura, Karimpur, Tehatta & adjacent areas Nadia, Paschimbangla |
| Rajshahi | Monigram, Bagha, Charghat, Shahtha, Yousufpur, Kajla, Belbukuria, Rajshahi town, Haripur, Godagari and adjacent area. | Jalangi, Gogadair Dhar, Laligolai, Azimganj, Bharanpur, Krishnapur, Raghunathganj, Aurangabad, Nittumala ,Bhusna, Baraigram , Zinjira , Agardari , Jamalpur, Murshidabad, Zinjira, Agardari, Jamalpur, Murshidabad, Paschimbango. |
