# Supplementary material for: Unveiling patterns and sociodemographic characteristics of drug trafficking in Dhaka city, Bangladesh
Source: PLoS One. 2026 Aug 10;21(8):e0349429. doi: 10.1371/journal.pone.0349429 (PMC13456272; doi:10.1371/journal.pone.0349429)
Supplement: Supplementary file 4 [file pone.0349429.s004.docx]

**S4 Appendix. Major border crossing routes based on Department of Narcotics Control.**

| **Regions** | **Country** | **Border crossing points** |
| --- | --- | --- |
| Western | India | Benapol, Dorshona, Dogachi, Parsha, Hiliy, Birol, Balubari, Banglabandha |
| Eastern | India and Myanmar | Latu, Ahammadabad, Akhaura, Kosbaa, Amratoly, Razapur, Braymmapara, Bibirbazar, Chaddagram, Suagazi, Mirja nagar, Ramghar, Barkal, Ukhia, Teknaf, Amratoly, Razapur, Braymmapara, Bibirbazar, Chaddagram, Suagazi |
| Northern | India | Tinbingha Corridor, Patgram, Mogholhat, Alatoly, Tambil |
