# Supplementary material for: Unveiling patterns and sociodemographic characteristics of drug trafficking in Dhaka city, Bangladesh
Source: PLoS One. 2026 Aug 10;21(8):e0349429. doi: 10.1371/journal.pone.0349429 (PMC13456272; doi:10.1371/journal.pone.0349429)
Supplement: Supplementary file 5 [file pone.0349429.s005.docx]

**S5 Appendix. Narcotics Seizures by DNC.**

| **Year** | **Heroin (kg)** | | **Weed (kg)** | | **Yaba (piece)** | |
| --- | --- | --- | --- | --- | --- | --- |
|  | **Case** | **Seizure** | **Case** | **Seizure** | **Case** | **Seizure** |
| 2018 | 50 | 0.505 | 921 | 390.638 | 843 | 497120 |
| 2019 | 49 | 0.308 | 1701 | 159.845 | 755 | 218051 |
| 2020 | 318 | 1.461 | 3155 | 690.633 | 1423 | 242823 |
| 2021 | 495 | 2.294 | 3727 | 1150.828 | 1368 | 4341272 |
| 2022 | 672 | 4.094 | 4448 | 2442.548 | 1603 | 828347 |

**Drug Seizures by DNC**

The Department of Narcotics Control's diligent surveillance over the past five years had yielded a significant uptick in drug seizures, a testament to their unwavering commitment to curtail the spread of narcotics (S5 Appendix.). The data portrayed a stark increase in heroin seizures, escalating from 0.505 kg to 4.094 kg, suggesting a heightened crackdown on this perilous opioid. Weed confiscations had multiplied, with a staggering 2442.548 kg seized in 2022, revealing the DNC's resolved in dismantling extensive trafficking networks (S5 Appendix.). Moreover, the surge in yaba seizures, from 497,120 pieces to 828,347, underscored the pervasiveness of synthetic drugs and the DNC's relentless efforts to eradicated their distribution (S5 Appendix.).

**References**

Department of Narcotics Control (n.d.). *Annual Drug Report 2022*. <https://dnc.gov.bd/>.
